# Whole-Genome Sequences of *Xanthomonas euvesicatoria* Strains Clarify Taxonomy and Reveal a Stepwise Erosion of Type 3 Effectors

**DOI:** 10.3389/fpls.2016.01805

**Published:** 2016-12-09

**Authors:** Jeri D. Barak, Taca Vancheva, Pierre Lefeuvre, Jeffrey B. Jones, Sujan Timilsina, Gerald V. Minsavage, Gary E. Vallad, Ralf Koebnik

**Affiliations:** ^1^UMR Interactions – Plantes – Microorganismes – Environnement, IRD-Cirad-Université MontpellierMontpellier, France; ^2^Department of Plant Pathology, University of WisconsinMadison, WI, USA; ^3^Faculty of Biology, Sofia University St. Kliment OhridskiSofia, Bulgaria; ^4^Pôle de Protection des Plantes, UMR Peuplements Végétaux et Bioagresseurs en Milieu Tropical, Cirad-Université de la RéunionSaint-Pierre, Ile de la Réunion, France; ^5^Department of Plant Pathology, University of FloridaGainsville, FL, USA; ^6^Gulf Coast Research and Education Center, University of FloridaWimauma, FL, USA

**Keywords:** *Xanthomonas euvesicatoria*, *Xanthomonas perforans*, comparative genomics, taxonomy, type III effectors, PIP box, HrpX regulon, cell wall degrading enzymes

## Abstract

Multiple species of *Xanthomonas* cause bacterial spot of tomato (BST) and pepper. We sequenced five *Xanthomonas euvesicatoria* strains isolated from three continents (Africa, Asia, and South America) to provide a set of representative genomes with temporal and geographic diversity. LMG strains 667, 905, 909, and 933 were pathogenic on tomato and pepper, except LMG 918 elicited a hypersensitive reaction (HR) on tomato. Furthermore, LMG 667, 909, and 918 elicited a HR on Early Cal Wonder 30R containing *Bs3*. We examined pectolytic activity and starch hydrolysis, two tests which are useful in differentiating *X. euvesicatoria* from *X. perforans*, both causal agents of BST. LMG strains 905, 909, 918, and 933 were nonpectolytic while only LMG 918 was amylolytic. These results suggest that LMG 918 is atypical of *X. euvesicatoria*. Sequence analysis of all the publicly available *X. euvesicatoria* and *X. perforans* strains comparing seven housekeeping genes identified seven haplotypes with few polymorphisms. Whole genome comparison by average nucleotide identity (ANI) resulted in values of >99% among the LMG strains 667, 905, 909, 918, and 933 and *X. euvesicatoria* strains and >99.6% among the LMG strains and a subset of *X. perforans* strains. These results suggest that *X. euvesicatoria* and *X. perforans* should be considered a single species. ANI values between strains of *X. euvesicatoria, X. perforans, X. allii, X. alfalfa* subsp. citrumelonis, *X. dieffenbachiae*, and a recently described pathogen of rose were >97.8% suggesting these pathogens should be a single species and recognized as *X. euvesicatoria*. Analysis of the newly sequenced *X. euvesicatoria* strains revealed interesting findings among the type 3 (T3) effectors, relatively ancient stepwise erosion of some T3 effectors, additional *X. euvesicatoria*-specific T3 effectors among the causal agents of BST, orthologs of *avrBs3* and *avrBs4*, and T3 effectors shared among xanthomonads pathogenic against various hosts. The results from this study supports the finding that T3 effector repertoire and host range are fundamental for the study of host—microbe interaction but of little relevance to bacterial speciation.

## Introduction

The genus *Xanthomonas* includes numerous phytopathogenic bacteria. While the physiological characteristics of *Xanthomonas* are quite homogeneous, biological diversity is evident in that the phytopathogenic xanthomonads cause disease on more than 400 hosts, ranging across 11 monocotyledonous, and 57 dicotyledonous families (Leyns et al., 1984). Although the genus *Xanthomonas* infects a wide variety of hosts that inhabit the full spectrum of ecological niches, individual strains usually have a narrow host range (Jacques et al., 2016). Historically, phytopathogenic bacteria nomenclature has been based on their host range. *Xanthomonas* that caused the same symptomology on the same host range were grouped into an infrasub-specific division, pathovar (Dye et al., 1980). However, *Xanthomonas* phylogeny based on nucleic acid analysis has begun to upend the rationale for phytobacterial systematics to be based on host range.

Classification of species within the genus *Xanthomonas* underwent major revision based on nucleic acid analysis. A comprehensive DNA-DNA hybridization study resulted in the recognition of 20 species (Vauterin et al., 1995). Subsequently, three additional species, *X. euvesicatoria, X. perforans*, and *X. gardneri*, that all cause bacterial spot of tomato (BST) were designated based on DNA-DNA hybridization (Jones et al., 2004). Using DNA-DNA hybridizations, repetitive element palindromic (Rep)-PCR, and amplified fragment length polymorphism (AFLP) genomic fingerprints the major phytopathogenic species of *Xanthomonas* were divided into six groups designated 9.1 to 9.6 (Rademaker et al., 2000, 2005; Ah-You et al., 2009). Collectively, these analyses confirmed nucleic acid distinctions among the causal agents of BST. *X. euvesicatoria* and *X. perforans* were placed in group 9.2 and *X. vesicatoria* with distinct rep-PCR fingerprints matched no other examined strains and was left outside any group. Each group was designated a distinct species, usually one for each group; however, within some groups, historical species nomenclature has retained, such as group 9.2 recognized as *X. euvesicatoria*, also includes *X. perforans, X. dieffenbachiae, X. alfalfae*, and several pathovars of *X. axonopodis*.

Further nucleic acid examination continues to erode phytobacterial systematics based on host range. Mutilocus sequencing analysis (MLSA) based on a very limited number of bacterial spot causing strains hypothesized that (i) *X. euvesicatoria* and *X. perforans*, and (ii) *X. gardneri* and *X. cynarae* likely are synonyms (Young et al., 2008). Using MLSA, average nucleotide identity (ANI), and DNA-DNA hybridizations Constantin et al. also concluded that *X. perforans* should be considered *X. euvesicatoria* (Constantin et al., 2016). More importantly, results from all of these nucleic acid analyses with an extensive collection of *X. dieffenbachiae* strains isolated from three distinct hosts support that these strains belong in four bacterial species, *X. euvesicatoria, X. citri, X. phaseoli*, and *X. axonopodis* independent of host range.

*Xanthomonas* phylogeny is not driven by host range and therefore its systematics should also be independent of the historical constraints commonly imposed on phytopathogenic bacteria. Evidence to support this supposition already exists in the case of the causal agents of bacterial spot of tomato and/or pepper. Bacterial spot is caused by four distinct species: *X*. *euvesicatoria, X. vesicatoria, X. perforans*, and *X. gardneri* (Jones et al., 2000). Among the four species, *X. euvesicatoria* and *X. gardneri* strains infect both tomato and pepper, *X. perforans* strains until recently only cause disease in tomato (Schwartz et al., 2015), and *X. vesicatoria* strains primarily infect tomato. Interestingly, a recent phylogenomic analysis of these four species included a *X. perforans* isolated from symptomatic pepper (Schwartz et al., 2015). The authors concluded that host range was determined by type 3 effector repertoire and to an extent AvrBsT limited it to tomato. Although this study included 67 genomes of *X. euvesicatoria, X. perforans*, and *X. gardneri*, collectively, they were all isolated from symptomatic tissue collected in the United States, a narrow geographical range when one considers that *X. euvesicatoria* has a worldwide distribution (Jones et al., 2005) and *X. perforans* and *X. gardneri* strains increasingly have been isolated in Canada (Cuppels et al., 2006), South America, and regions bordering the Indian Ocean (Bouzar et al., 1996, 1999; Hamza et al., 2010). Although this recent trove of genomes of *X. euvesicatoria, X. perforans*, and *X. gardneri* is a useful set to examine questions of pathogen population structure and recent pathogenicity factor changes among some of the causal agents of bacterial spot of pepper and tomato, the available sequenced genomes remain temporally and geographically biased.

In this study we sequenced five *Xanthomonas euvesicatoria* strains isolated from three continents (Africa, Asia, and South America) to provide a set of representative genomes for further comparative analyses with the available sequenced strains isolated from the United States, the Balkan Peninsula, and Italy. Strains were isolated from either symptomatic *Capsicum* or *Lycopersicon* when recorded. We broadly analyzed nucleic acids and gene content of the strains we sequenced as well as all the available *X. euvesicatoria* and *X. perforans* sequenced strains. By ANI, we examined the phylogeny of *X. euvesicatoria, X. perforans, X. allii, X. alfalfa* subsp. citrumelonis, and *X. dieffenbachiae*, members of Rademaker group 9.2. By comparing multiple members of Rademaker group 9.2, we provide a unique, integrated phylogeny of *X. euvesicatoria* independent of host range. We also provide evidence based on genomic sequencing of a xanthomonad isolated from rose that should be placed in *X. euvesicatoria* as a new pathovar. With the genome sequences of these geographically and temporally diverse set of *X. euvesicatoria* LMG strains, as well as the *X. euvesicatoria* pv. rosa strain, T3 effector evolution has been examined and sets a foundation for future hypothesis-driven research.

## Results/methods/discussion

### Phenotypic evaluation

Five strains of *X. euvesicatoria* were selected from the Belgium Co-ordinated Collection of Micro-organisms/LMG (http://bccm.belspo.be/about-us/bccm-lmg) for inquiry to expand our understanding about the causal agent of bacterial spot of tomato and pepper. Strains LMG 918 and LMG 933 were isolated from *Capsicum frutescens* from India in 1957 and Brazil, respectively. Strain LMG 909 was isolated from *Capsicum* sp. from the Ivory Coast in 1979. Strain LMG 667 was isolated from *Lycopersicon esculentum*, origin unknown and strain LMG 905 was isolated in India, host unknown. Since these strains were isolated from various hosts, we performed pathogenicity tests on tomato and pepper by infiltration using *X. euvesicatoria* strain 85-10 as a positive control. Overnight cultures were grown using in nutrient broth, cells were pelleted by centrifugation, and pellets were resuspended in water. Plant leaves were infiltrated by needleless syringe containing a water-bacterial suspension of 10^8^ CFU/ml or water as a control. All the strains caused typical bacterial spot lesions on tomato (Bonny Best) and pepper (Early Cal Wonder, ECW), except LMG 918 which was only pathogenic on pepper. Race analysis on pepper with ECW-30R which contains *Bs3* showed LMG 667, 909, and 918 elicited a hypersensitive reaction (HR) and thus contain *avrBs3.* LMG strains 667, 905, 909, and 933, but not 918, elicited a HR on ECW-20R which contains *Bs2*. Race analysis on tomato with Hawaii 7998 which has resistance that interacts with *avrRxv* resulted in a HR for LMG strains 667, 905, 909, and 933. These results suggest differences in the functional type 3 effectors among the LMG strains which we examined following genome sequencing.

Historically, BST pathogens, *X. euvesicatoria* and *X. perforans*, have been differentiated biochemically by pectate utilization and starch hydrolysis (Stall et al., 1994). In general, *X. euvesicatoria* strains are neither pectolytic nor amylolytic. All the strains, LMG 667, 905, 909, 918, and 933, failed to cause a depression around a bacterial colony on CVP medium by 48 h suggesting they are nonpectolytic. Surprisingly, LMG 918 was amylolytic as it and the positive control, *X. perforans* 485, displayed copious growth and a turbid halo around each colony grown for 48 h on nutrient agar supplemented with 1.5% soluble starch following flooding of plates with Lugol's iodine solution. The other LMG strains were nonamylolytic. These results suggest that LMG strains 667, 905, 909, and 933 react similar to *X. euvesicatoria* strains. In contrast, LMG 918 is different than the typical *X. euvesicatoria* or *X. perforans* strains, since *X. perforans* strains are strongly pectolytic and amylolytic (Jones et al., 2004). Neither these biochemical tests nor pathogenicity tests can confirm the species of all the LMG strains tested.

### Genome sequencing and annotation

The genomes of the five LMG strains, 667, 905, 909, 918, and 933, were sequenced using the Illumina Hi-Seq2500 platform (Fasteris SA, Switzerland). The shotgun sequencing yielded between 1,917,527 and 3,384,562 100-bp paired-end reads (474–966 Mb), with insert sizes ranging from of 250 bp to 1.5 kb (Table 1). Draft genome sequences were assembled using the Edena algorithm v3.131028 (Hernandez et al., 2014), yielding between 375 and 498 contigs ≥200 bp (N_50_ between 19,253 and 29,903 bp) with 65–165 × coverage. Contigs were annotated with GeneMarkS + release 2.9 (revision 452131) (Borodovsky and Lomsadze, 2014), as implemented in the NCBI Prokaryotic Genome Annotation Pipeline (http://www.ncbi.nlm.nih.gov/genome/annotationprok/), which predicted between 4539 and 4879 genes per genome. These whole genome shotgun projects have been deposited at DDBJ/EMBL/GenBank under the accession no. JTEH00000000 to JTEL00000000 and the raw sequence reads are accession nos. SRR4712532, SRR4713555, SRR4714146, SRR4714148, and SRR4714149.

**Table 1:** **Whole-genome sequence details**.

**Strain**	**BioProject (PRJNA)**	**BioSample (SAMN)**	**GenBank acc. no**.	**Paired-end reads**	**Yield (Mb)**	**Coverage**	**Contigs ≥200 bp**	**Size (bp)**	**N_50_ (bp)**	**Genes**
LMG 667	267081	03177540	JTEH00000000	1,917,527	479	69 x	375	5,396,926	29,903	4879
LMG 905	267089	03177541	JTEI00000000	2,259,823	565	73 x	399	5,157,086	24,007	4620
LMG 909	267084	03177542	JTEJ00000000	1,895,699	474	65 x	380	5,092,005	23,348	4539
LMG 918	267090	03177543	JTEK00000000	1,950,905	488	75 x	407	5,110,294	22,691	4563
LMG 933	267091	03177544	JTEL00000000	3,864,562	966	165 x	498	5,063,804	19,253	4557
GEV-Rose-07	342189	05750688	MIKD00000000	341,918	72.9	16.6 x	499	4,970,862	20720	4510

na, not available at this time.

The genome of GEV-Rose-07 strain was sequenced using Illumina Miseq platform (Interdisciplinary Center for Biotechnology Research, University of Florida). The sequences yielded 341,918 reads of average 241-bp paired-end reads (72.86 Mb). Draft genome was assembled using CLC Genomics Workbench v5, yielding 499 contigs ≥ 500 bp (*N*_50_ = 20,720 bp) with 16.6 × coverage. The assembled sequence was annotated using IMG/JGI platform, with the gene prediction underway. The genome has been deposited at GenBank under the accession number MIKD00000000 and the raw sequence read is accession no. SRR4457940.

### Hydrolytic enzymes related to taxonomy

To gain an understanding of the biochemical differentiation among LMG 918 and other *X. euvesicatoria* strains, we examined the genome sequences for amylases using the Carbohydrate-Active enZymes Database (CAZy; www.cazy.org). We found five putative polysaccharide lyases from three families in *X. euvesicatoria* 85-10, *X. perforans* 4P1S2, and the LMG strains 667, 905, 909, 918, and 933. Three CAZy families, GH13, GH14, and GH57, contain amylases. Amylases from the GH13 family were identified in *X. euvesicatoria* strain 85-10. Of the seven genes that were found, five encode cytoplasmic proteins with regions of similarity to alpha-amylases (annotated as putative alpha-amylase family protein, putative trehalose synthase, maltooligosyltrehalose synthase, alpha-glucosidase, and sucrose hydrolase). Two genes encode secreted proteins, annotated as putative alpha-amylase and cyclomaltodextrin glucanotransferase precursor. Interestingly, the putative alpha-amylase in *X. euvesicatoria* strain 85-10 contains a frameshift. A comparison of the *X. euvesicatoria* and *X. perforans* genome sequences revealed that all but three *X. euvesicatoria* strains but none of the *X. perforans* strains contain this frameshift (XCV0850/XCV0849). These results suggest that *X. euvesicatoria* strains LMG 918, Xe 259, and Xe 315 could be amylolytic which was confirmed in this study for LMG 918.

### Comparison of genome sequences

Since phenotypic evaluation separated *X. euvesicatoria* LMG 918 from the other LMG strains and previously described *X. perforans* and *X. euvesicatoria* strains, we executed an extensive analysis of the sequenced genomes of both species. We compared portions of seven housekeeping genes (4722 bp in total), *atpD, dnaK, efp, glnA, gyrB, lepA*, and *rpoD*, from all publicly available genomes of *X. euvesicatoria* and *X. perforans*, as well as the strains sequenced in this study. Polymorphisms were rare. In total, seven haplotypes were found among the 68 strains. A large *X. euvesicatoria* haplotype group (H1) consists of strains from each geographical region examined with a total of 31 strains (Table 2). Thus, sequences of these essential genes from four of the newly characterized *X. euvesicatoria* strains in this study were identical to the bulk of strains from the United States. *X. euvesicatoria* strains 259 and 315 were separated from H1 by one SNP. A large *X. perforans* group (H6) consists of 29 strains. The sequence of strain *X. perforans* 4P1S2 contains five “N” which introduce frameshifts in the genes *dnaK* and *glnA*; removal of the Ns results in a sequence identical to the other members of H6. *X. perforans* strain Xp17-12 has one SNP with respect to H6. *X. perforans* strains 4–20 and 5–6 are more similar to H1 than H6 while LMG 918, which lacks 7 bp in the *atpD* gene due to its split into two contigs, is as similar to H1 as H6. Recently, a core protein-coding genome phylogenetic analysis identified a division among these *X. perforans* strains separating them into three groups (Schwartz et al., 2015), which grouped strains Xp 17-2, Xp 4-20, and Xp 5-6 together. Furthermore, their own SNP analysis including 22,105 SNPs in the *X. perforans* genomes compared to the reference *X. axonopodis* pv. citri strain 306 grouped these strains (Xp 17-2, Xp 4-20, and Xp 5-6) together tightly with strains we separated into H6, e.g., Xp15-11, Xp11-2, and Xp18-15. In general, the strains analyzed from the United States are relatively young, with the oldest isolated from 1998 while the LMG strains we sequenced in this study are significantly older, when isolation dates are known, such as LMG 918 and LMG 909 were isolated in 1957 and 1979, respectively. Neither geographic nor temporal factors appear to influence divisions among these strains based on SNP analysis. Results from the comparison of these niche independent genes suggest that although a few polymorphisms exist among *X. euvesicatoria* and *X. perforans* strains, separating the strains into distinct species is poorly supported by examination of a broad collection of strains.

**Table 2:** **SNP analysis of seven housekeeping genes, ***atpD, dnaK, efp, glnA, gyrB, lepA***, and ***rpoD***, separating all publicly available genomes of ***Xanthomonas euvesicatoria*** and ***X. perforans*** strains into haplotypes (H#)**.

	**H1**	**H2**	**H3/H4**	**H5**	**H6**	**H7**
Reference	Xe 85-10^a^				Xp 91-118^b^	
Balkan^c^	66b, 83M					
Brazil	LMG 933					
India	LMG 905			LMG 918		
Italy^d^					Xp 4P1S2	
Ivory Coast	LMG 909					
United States^e^	Xe 181 Xe 199 Xe 206 Xe 329 Xe 354 Xe 376 Xe 455 Xe 490 Xe 515 Xe 526 Xe 586 Xe 678 Xe 679 Xe 681 Xe 683 Xe 684 Xe 685 Xe 689 Xe 695 Xe F4-2 Xe G4-1 Xe H3-2 Xe L3-2	Xe 259 Xe 315	Xp 4-20 Xp 5-6		Xp GEV839 Xp GEV872 Xp GEV893 Xp GEV904 Xp GEV909 Xp GEV915 Xp GEV917 Xp GEV936 Xp GEV940 Xp GEV968 Xp GEV993 Xp GEV1001 Xp GEV1026 Xp GEV1044 Xp GEV1054 Xp GEV1063 Xp TB6 Xp TB9 Xp TB15 Xp 3-15 Xp 4B Xp 7-12 Xp 8-16 Xp 9-5 Xp 10-13 Xp11-2 Xp 15-11 Xp 18-15 Xp 2010	Xp 17-12
Unknown	LMG 667 LMG 27970					

Strains previously sequenced

a *Thieme et al. (2005)*,

b *Potnis et al. (2011)*,

c *Vancheva et al. (2015)*,

d Torelli et al. (2015), and

e Schwartz et al. (2015).

To determine the validity of separating *X. euvesicatoria* and *X. perforans* into two species, ANI was calculated using JSpecies (Richter and Rosselló-Móra, 2009) for all the LMG strains sequenced in this study, *X. euvesicatoria* strains 66b and 83M, isolated from symptomatic *Capsicum annuum* from Bulgaria in 2012 and Macedonia in 2013, respectively (Vancheva et al., 2015), and *X. euvesicatoria* 85-10, the reference strain for genomics (Thieme et al., 2005). ANI values among all these *X. euvesicatoria* strains were >99.1% (Table 3). A BLAST-based comparison of a subset of *X. perforans* strains isolated in the United States and separated into three groups by Schwartz et al. based on ML analysis based on partitioned analysis by codon position, revealed ANI values between 99.62 and 99.7%. Neither SNP analysis, presence (or absence) of specific type 3 (T3) effectors, nor ANI support meaningful divisions within *X. perforans*. These results suggest that differences within *X. perforans* genomes are not relevant to bacterial infrasub-specific division phylogeny. We found ANI values between the same subset of *X. perforans* strains and *X. euvesicatoria* strains (85-10, 66b, 83M, and all the LMG strains described herein) were >98.1% (Table 3). Since ANI is considered the new standard for species definition, these results suggest that the strains sequenced in this study are *X. euvesicatoria* and that *X. perforans* strains should be considered *X. euvesicatoria*, similar to the findings of others (Young et al., 2008; Constantin et al., 2016).

**Table 3:** **Average nucleotide identity (ANI) values for a two-way comparison between two genomes of ***Xanthomonas euvesicatoria*** and/or ***X. perforans*** strains**.

	**Xe 85-10**	**Xe 66b**	**Xe 83M**	**LMG 667**	**LMG 905**	**LMG 909**	**LMG 918**	**LMG 933**	**Xp GEV968**	**Xp 91-118**	**Xp 17-12**	**Xp 5-6**	**Xp 4B**	**Xp TB6**	**Xp TB15**
Xe 85-10	–	99.83	99.86	99.85	99.78	99.84	99.28	99.84	98.43	98.48	98.54	98.44	98.40	98.60	98.58
Xe 66b	99.85	–	99.82	99.80	99.39	99.86	99.27	99.93	98.31	98.39	98.38	98.35	98.26	98.42	98.44
Xe 83M	99.90	99.84	–	99.93	99.92	99.93	99.20	99.93	98.32	98.37	98.40	98.38	98.26	98.40	98.46
LMG 667	99.80	99.71	99.85	–	99.31	99.91	99.10	99.91	98.24	98.34	98.30	98.30	98.21	98.33	98.39
LMG 905	99.88	99.48	99.92	99.48	–	99.91	99.23	99.92	98.45	98.48	98.52	98.43	98.39	98.52	98.52
LMG 909	99.88	99.90	99.93	99.99	99.92	–	99.19	99.99	98.35	98.42	98.41	98.42	98.32	98.43	98.50
LMG 918	99.32	99.33	99.23	99.23	99.28	99.23	–	99.30	98.40	98.48	98.44	98.41	98.37	98.44	98.44
LMG 933	99.90	99.96	99.93	99.99	99.92	99.99	99.26	–	98.44	98.46	98.50	98.41	98.38	98.52	98.51
XpGEV968	98.49	98.40	98.40	98.35	98.46	98.36	98.39	98.46	–	99.81	99.79	99.87	99.85	99.80	99.78
Xp 91-118	98.55	98.51	98.44	98.46	98.55	98.45	98.52	98.54	99.81	–	99.74	99.91	99.74	99.74	99.82
Xp 17-12	98.50	98.43	98.39	98.34	98.53	98.38	98.40	98.48	99.72	99.67	–	99.67	99.62	99.66	99.64
Xp 5-6	98.36	98.33	98.34	98.29	98.38	98.37	98.31	98.36	99.77	99.83	99.64	–	99.87	99.72	99.74
Xp 4B	98.36	98.28	98.26	98.24	98.35	98.26	98.26	98.36	99.79	99.75	99.66	99.90	–	99.74	99.73
Xp TB6	98.56	98.49	98.37	98.37	98.50	98.37	98.42	98.49	99.71	99.67	99.62	99.75	99.72	–	99.97
Xp TB15	98.46	98.43	98.40	98.39	98.41	98.40	98.33	98.42	99.66	99.65	99.59	99.72	99.66	99.91	–

Since the ANI values between *X. euvesicatoria* and *X. perforans* strains were above the 95–96% transition zone, above which strains are considered to be a taxonomically prokaryotic species (Konstantinidis and Tiedje, 2005), we did a BLAST-based comparison of members of the Rademaker group 9.2, *X. euvesicatoria, X. perforans, X. allii, X. alfalfae* subsp. *citrumelonis*, and *X. dieffenbachiae*, as well as a recently described pathogen of rose (Huang et al., 2013). This comparison revealed ANI values between 99.11 and 97.83% among the compared genomes (Table 4). These results are significantly above the transition zone and suggest that members of Rademaker group 9.2 are a single species and should be recognized as *X. euvesicatoria*, regardless of host range.

**Table 4:** **Average nucleotide identity (ANI) values for a two-way comparison between two genomes of ***Xanthomonas*** species assigned to Rademaker group 9.2**.

	***X. euvesicatoria* 85-10**	***X. perforans* 911-118**	***X. axonopodis* pv. allii CFBP6369**	***X. alfalfa* subsp. *citrumelonis* F1**	***X. dieffenbachiae* LMG 12749**	***X. axonopodis* pv. rose GEV-07**
*X. euvesicatoria* 85-10	–	98.48	98.14	98.47	98.35	97.79
*X. perforans* 911-118	98.55	–	98.10	98.71	98.80	98.49
*X. axonopodis* pv. allii CFBP6369	98.23	98.64	–	98.62	98.75	98.09
*X. alfalfa* subsp. *citrumelonis* F1	98.47	98.64	98.60	–	98.59	98.27
*X. dieffenbachiae* LMG 12749	98.42	98.75	98.77	98.64	–	98.37
*X. axonopodis* pv. rose GEV-07	98.72	98.51	99.11	98.62	97.83	–

Our results add to the mounting evidence that *Xanthomonas* phylogeny is not driven by host range. Nonetheless, the systematics of phytobacterial pathogens which reflects host range is valuable to the scientific community as well as to a broader audience, such as regulators. Our phylogenetic analysis support designation of the recently described pathogen of rose as *X. euvesicatoria*, and yet we advocate for the use of pathovar rosa for those strains which share the same host range (Huang et al., 2013).

### Type III effectors

**Figure 1:**
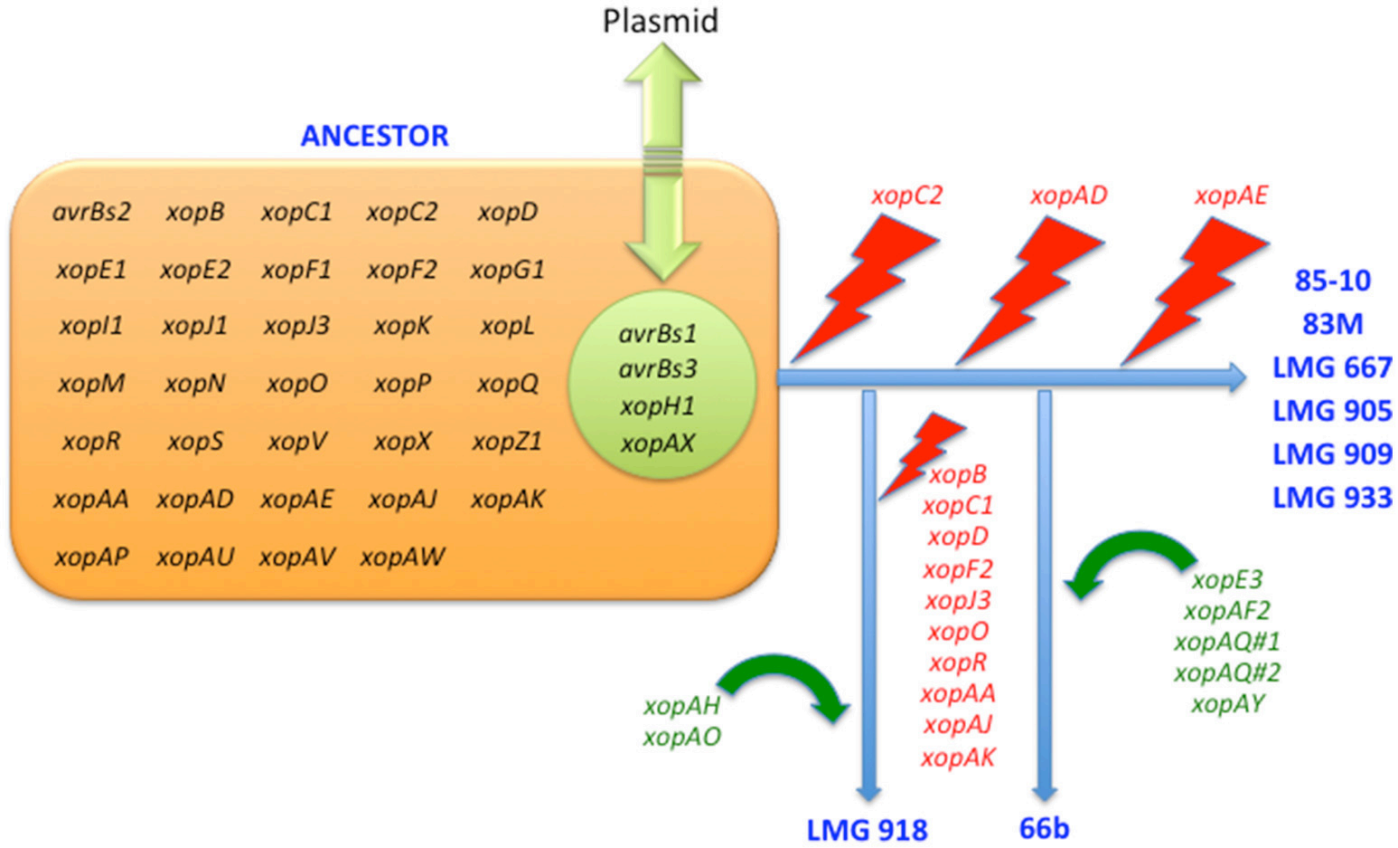
**Erosion and acquisition of type 3 effectors among ***Xanthomonas euvesicatoria*** strains**.

Pathogenicity on specific hosts by xanthomonads has been attributed to the presence or absence of specific T3 effectors (Schwartz et al., 2015). Previously, a group of T3 effectors were identified as a set core in the four *Xanthomonas* species which cause BST (Potnis et al., 2011). The LMG strains sequenced in this study possess each of these T3 effectors with the exception of XopAD (Table 5). The *xopAD* gene, which encodes a SKWP repeat protein, was found to be intact in *X. perforans*, the rose isolate, and LMG 918, but has several conserved internal stops in the other strains, suggesting that they all originate from a common ancestor. Similar findings were observed with *xopC2*, which shares the same inactivating frameshift mutations in all the LMG strains, the two strains from the Balkan Peninsula, and the *X. euvesicatoria* reference strain 85-10, and with *xopAE*, which has a conserved frameshift in strains 85-10, 83M, and all LMG strains except LMG 918. A stepwise erosion process of *xopAD*Ω, *xopC2*Ω, and *xopAE*Ω is relatively ancient to the species as suggested by their G+C content for strain 85-10, 66.9, 60.9, and 63.7%, respectively (Figure 1). We speculate that LMG 918 and *X. perforans* strains 91-118 and 4P1S2 may have a shared ancestor separate from *X. euvesicatoria* strains 85-10, 66b, 83M, and LMG 667, 905, 909, and 933 that started to accumulate mutations in *xopAD.* The ancestor shared by *X. euvesicatoria* strains 85-10, 66b, 83M, and LMG 667, 905, 909, and 933 accumulated inactivating mutations in *xopC2*. Later, the lineage with the inactivated *xopC2* incurred a frameshift mutation in *xopAE*. This scenario is an example of T3 effector repertoire evolution which may influence host specificity.

**Table 5:** **Distribution of type 3 effectors not found in every ***Xanthomonas*** strain sequenced in this study**.

Effector	**Xe 85-10**		**Xe 83M**	**LMG 667**	**LMG 905**	**LMG 909**	**LMG 933**	**LMG 918**	**Xe 66b**	**Xp 91-118**	**Xp 4P1S2**	**R07**
XopB	XCV0581	+	+	+	+	+	+	IS	+	−	−	−
XopC1	XCV2435	+	+	+	+	+	+	−	+	−	−	−
XopC2	XCV1239 XCV1238 XCV1237	Ψ (fs)	Ψ (fs)	Ψ (fs)	Ψ (fs)	Ψ (fs)	Ψ (fs)	Ψ (fs)	Ψ (fs)	+	+	+
XopD	XCV0437	+	+	+	+	+	+	Ψ (fs)	+	+	+	−
XopE1	XCV0294	+	+	+	+	+	+	+	+	+	+	+
XopE2	XCV2280	+	+	+	+	+	+	+	+(sc)	−	−	+
XopE3		−	−	−	−	−	−	+	+	−	IS	−
XopF1	XCV0414	+	+	+	+(sc)	+	+	+	+	+	+(ag)	+
XopF2	XCV2942	+	+	+	+	+	+	Ψ (fs/stop)	+(sc)	+	+	+
XopG1	XCV1298	+	−	−	−	−	−	−	−	−	−	−
XopH1	XCVd0105	+	−	−	−	−	−	−	−	−	−	−
XopI3		−	−	−	−	−	−	−	−	+	+	−
XopI4		−	−	−	−	−	−	−	−	+	+(ag)	−
XopJ1	XCV2156	+	+	+	+	+	+	+	+	−	−	−
XopJ2		−	−	−	−	−	−	−	−	−	+(sc)	−
XopJ3	XCV0471	+	+	+	+	+	+	IS	+	−	−	−
XopJ4		−	−	−	−	−	−	−	−	+	+	−
XopO	XCV1055	+	Ψ (stop)	Ψ (fs)	+	+	+	IS	Ψ (fs)	−	−	−
XopP#2		−	−	−	−	−	−	−	−	+	+(ag)	+
XopR	XCV0285	+	+	+	+	+	+	Ψ (fs)	+	+	+	+
XopW		−	−	−	−	−	−	−	−	−	−	Ψ (fs)
XopAA	XCV3785	+	+(sc)	+	+	+	+	Ψ (fs)	+	−	−	−
XopAD	XCV4315 XCV4314 XCV4313	Ψ (stop)	Ψ (stop)	Ψ (stop)	Ψ (stop)	Ψ (stop)	Ψ (stop)	+(sc)	Ψ (stop)	+	+(ag)	+(sc)
XopAE	XCV0409 XCV0408	Ψ (fs)	Ψ (fs)	Ψ (fs)	Ψ (fs)	Ψ (fs)	Ψ (fs)	+	+	+	+(ag)	+
XopAF1		−	−	−	−	−	−	−	−	+	+(ag)	+
XopAF2		−	−	−	−	−	−	+	+	−	−	−
XopAH		−	−	−	−	−	−	+	−	−	−	−
XopAJ	XCV4428	+	+	+	+(sc)	+	+	IS	+	−	−	+
XopAK	XCV3786	+	+	+	+	+	+	Ψ (fs)	+	+	+	+
XopAO		−	−	−	−	−	−	+	−	−	−	−
XopAQ#1		−	−	−	−	−	−	+	+	−	+	−
XopAQ#2		−	−	−	−	−	−	+	+	−	−	−
XopAR		−	−	−	−	−	−	−	−	+	+(ag)	−
XopAX	XCVd0086	+	−	−	−	−	−	−	+	−	−	−
XopAY		−	−	−	−	−	−	+	+	−	+(sc)	−

sc, gene on split contigs; ag, assembly gap; Ψ, pseudogene due to IS, fs, or stop; IS, gene disrupted by an insertion sequence; fs, frameshift; or stop, early stop codon.

The stepwise erosion process of some T3 effector genes as described above appears to be compensated by additional, lineage-specific T3 effectors. Strains 66b and LMG 918 share five T3 effectors, which are not present in the other LMG strains, 83b, 85-10, or *X. euvesicatoria* pv. rosa (Table 5). However, it is widespread in field isolates of *X. euvesicatoria* from the United States (Schwartz et al., 2015), listed in Table 2. An inactivated variant, containing an IS element, was found in the genome of *X. perforans* 4P1S2. The low G+C content of this gene (59.0%) suggests a more recent acquisition. XopE3 belongs to the XopE class of T3 effectors and has close homologs in *X. axonopodis, X. citri, X. fuscans, X. arboricola, X. cassavae*, and *X. campestris*. Next to *xopE3*, facing each other, we found a homolog of *xopAQ* (G+C content of 56.6% with strain 85-10). A paralog of *xopAQ* with an even lower G+C content (53.8% with strain 85-10) is upstream of the T3 effector gene *xopAY* (G+C content of 52.6%). XopAY, which is related to HopW1 from *Pseudomonas syringae* (Lee et al., 2008), was first found in several *X. translucens* genomes but is also present in *X. bromi, X. hyacinthi*, and *X. vasicola*, all infecting monocots. The finding that this T3 effector is also present in strains 66b, LMG 918 and the *X. perforans* strain 4P1S2 suggests a function in dicotyledonous plants as well. XopAQ is related to a T3 effector (Rip6/Rip11) from *Ralstonia solanacearum* (Mukaihara et al., 2010). The two *xopAQ* paralogs have homologs in *X. arboricola, X. citri*, and *X. gardneri*, where it was first described in *Xanthomonas* (Potnis et al., 2011). XopAF2, another T3 effector specific to only *X. euvesicatoria* strains 66b and LMG 918, is related to the widespread HopAF1 effector from *P. syringae*, which suppresses plant immunity by targeting methionine recycling to block ethylene induction (Washington et al., 2016). Close homologs are currently only found in *X. citri* and *X. fuscans*, two species that were placed in Rademaker group 9, and in strains of *X. arboricola*.

Although we hypothesize that strains 66b and LMG 918 arose from separate lineages, they share T3 effectors absent in the reference strain 85-10 which we suppose shares a common ancestor with 66b. All three 66b/LMG 918-specific loci are syntenic in both strains. For the *xopE3*-*xopAQ* locus, we found a corresponding region in the reference strain 85-10, harboring XCV2439 and XCV2440 (*hpaJ*), in one of the two flanking sequences (2 kb). Similarly, for the *xopAF2* locus, only one of the two flanking sequences has a counterpart in strain 85-10, encoding the two cointegrate resolution proteins S and T (XCV2438 and XCV2437). This close vicinity of the two loci is striking, and their vicinity to cointegrate resolution genes suggests a recombination-based acquisition mechanism, either from a plasmid or a transposon. Finally, the *xopAQ*-*xopAY* loci are also syntenic in strains 66b and LMG 918, and no homologous region is found in the reference strain 85-10. These data suggest that determination of ancestral lineage based on T3 effector repertoires may be futile whereas individual T3 effector lineage may be possible and informative to host—microbe interactions.

We also identified additional T3 effectors shared among all the LMG strains, *X. euvesicatoria* 85-10, and *X. perforans* 91-118 and 4P1S2, XopE1, XopI1, XopM, XopP#1, XopS, XopV, XopAP, XopAU, XopAV, and XopAW (Table 5). Surprisingly, all of these T3 effectors, as well as the ones identified as shared among BST pathogens previously (Potnis et al., 2011), were also found in *X. euvesicatoria* pv. rose. These T3 effectors must not be responsible for pathogenicity of tomato since *X. euvesicatoria* pv. rose was shown to be non-pathogenic to tomato (Huang et al., 2013). Taken together, these findings suggest that these T3 effectors are common among members of the Rademaker 9.2 clade and may be shared by a common ancestor but they are not involved in host determination.

Species-specific T3 effectors have also been previously identified for *X. perforans* (XopC2, XopJ4, XopAF, and XopAE) and *X. euvesicatoria* (AvrBs1, XopC1, XopJ1, XopJ3, XopO, XopAA, and XopAI) (Potnis et al., 2011). With the exception of LMG 918, in general, the LMG strains sequenced here have the classic *X. euvesicatoria*-specific T3 effectors previously identified, except AvrBs1. *X. euvesicatoria* strain 85-10 also includes XopH1, which like AvrBs1 is encoded next to each other on a plasmid, and XopG1, which is also unique to strain 85-10. The *xopG1* (XCV1298) gene is located between insertion elements IS*xac2* (XCV1297/XCV1296) and IS*1477* (XCV1301/XCV1300) upstream of another 85-10-specific gene, XCV1299, which encodes a putative secreted protein.

Using the LMG strains 667, 905, 909, and 933 and *X. euvesicatoria* 85-10, 66B, and 83M sequences, we identified additional *X. euvesicatoria*-specific T3 effectors, XopB, XopD, XopE2, XopF1, XopF2, XopK, XopL, XopN, XopQ, XopR, XopX, XopAA, XopAJ, and XopAK. LMG 918 has a single *X. euvesicatoria*-specific T3 effector, XopC1 and a single *X. perforans*-specific effector, XopAE. Since we found that LMG 918 infects pepper, a reduced number of *X. euvesicatoria*-specific T3 effectors is surprising. None of the remaining LMG strains have any of the *X. perforans*-specific effectors and furthermore, they only shared T3 effectors with *X. perforans* strains 91-118 and 4P1S2 that were also shared with *X. euvesicatoria* strains. Previously XopF1 has been hypothesized to be a pathogenicity determinant of *Xanthomonas* in tomato. However, we identified XopF1 in *X. euvesicatoria* pv. rose and this strain was shown to not infect tomato (Huang et al., 2013). Collectively, these results emphasize the conclusion that T3 repertoires may be poor determinants of phylogeny and inconclusive for speciation.

A pathogen population shift in *X. perforans* has been observed in Florida from tomato race 3 to race 4 due to null mutations in the *xopAE*/*avrXv3* gene. This shift has been noted as significant since the *X. perforans* reference strain 91-118, isolated in 1991, has XopAE while the *X. euvesicatoria* reference strain 85-10 does not have this T3 effector (Potnis et al., 2011). XopAE is a translational fusion of *hpaG* and *hpaF*. *X. euvesicatoria* strain 85-10, and *X. euvesicatoria* strains isolated in the United States before 1997, have separate *hpaG* and *hpaF* genes. We hypothesize that the LMG strains sequenced in this study would also have separate *hpaG* and *hpaF* genes and thus, lack XopAE. We found that only strain LMG 918 had an intact XopAE, similar to *X. euvesicatoria* 66b, *X. perforans* 91-118 and 4P1S2, and *X. euvesicatoria* pv. rose. This result is interesting since LMG 918 was isolated 40 years before XopAE appeared in the *X. perforans* population in the United States. All of the *X. euvesicatoria* strains isolated after 1997 from the United States also possess XopE3 (Schwartz et al., 2015); again, we only found XopE3 in LMG 918 and strain 66b from the Balkan Peninsula. The lag in appearance of XopE3 in the United States *X. euvesicatoria* population suggests a later introduction of *X. euvesicatoria* XopE3-containing strains into the United States or XopE3 arose multiple times in *X. euvesicatoria*.

A striking observation concerns the *xopO* gene, which suffered from mutational inactivation by at least four different events. The 211-codon gene has a frameshift at codon 14 in strain 66b, another frameshift at codon 73 in strain LMG 667, an early stop codon at codon 77 in strain 83M, and an IS element insertion with an 8-bp target site supplication at codon 136 in strain LMG 918. These multiple inactivation events that were retained suggest an advantage for getting rid of this protein, and it is tempting to speculate that XopO might be recognized as an avirulence factor by a resistance gene.

Targeting of T3 effectors to specific intracellular structures has been shown to affect the function. For instance, post-translational modification involving the covalent attachment of a lipid moiety (e.g., myristate or palmitate) has been shown to target proteins to the cytoplasmic membrane; this targeting is facilitated by a simple sequence motif at the N terminus of the polypeptide chain (Thieme et al., 2007). Prediction of such motifs in T3 effectors in the newly sequenced strains, using the CSS-Palm suite, reveals potential myristoylation/palmitoylation motifs for XopE1, XopE2, XopJ1, XopJ3, XopAF2, XopAH, XopAK, and XopAQ. Notably, we identified such a motif, MGNC, within the polypeptide chain of XopS (Schulze et al., 2012). Scrutinizing the 5′ region of the gene suggests that the corresponding ATG translational start codon is 15 nucleotides downstream of the annotated start codon in the *X. euvesicatoria* reference strain 85-10. This alternative start site would be accompanied by a well-defined Shine-Dalgarno sequence (GGAG) eight nucleotides upstream of the start codon. Another strongly predicted myrostoylation/palmitoylation motif, MGLC (preceded by a GGAG Shine-Dalgarno sequence six nucleotides upstream of the ATG start codon), is encoded 317 bp in front of the annotated ORF for XopB, only 29 bp downstream of a consensus PIP box-regulated promoter (see below). It will be interesting to analyze whether this candidate translational start site is functional, which would lead to the synthesis of a 24-amino acid peptide.

### Predicted HrpX regulons

Many, if not most, T3 effector genes are co-regulated with the *hrp* genes that encode the T3 secretion machinery (Roux et al., 2015). Moreover, additional virulence factors, such as cell-wall degrading enzymes, are co-regulated as well. All these genes are under control of a key regulatory protein of the AraC family, HrpX, which binds to a conserved sequence element in their promoter regions, the so-called PIP box (Koebnik et al., 2006). We therefore analyzed the six new genome sequences for the presence of the promoter motif TTCGB-N_15_-TTCGB-N_30−32_-TYNNNT (B represents C, G, or T; Y represents C or T). Using this conservative query, 24 putative PIP box-regulated promoters were found in the *X. euvesicatoria* strains and 18 were found in the *X. euvesicatoria* pv. rosa strain (Table 6). Most of the identified promoters corresponded to known HrpX-dependent genes, such as the *hrp*/*hpa* genes, genes for T3 effectors or cell-wall degrading enzymes (Noël et al., 2001; Jacobs et al., 2015). In addition, we found genes for three putative secreted proteins (XCV2568, XCV2729, and XCV4424) and a gene annotated to encode an anthranilate synthase component to be under control of HrpX, as previously experimentally confirmed for strain 85-10 (Noël et al., 2001; Koebnik et al., 2006). Two of these genes are not present in the rose isolate, neither did we find the T3 effector genes *xopB, xopC1, xopJ1*, and *xopAA*, which explains the smaller number of predicted PIP box-regulated promoters in this strain. We found only one predicted promoter that was oriented opposite to an annotated gene (XCV1852), a case that might represent a false positive. We would like to emphasize, however, that the *hrpX* regulon is certainly larger than the set of 18 to 24 genes discussed above. It is well known that mismatches to the PIP box consensus sequence can be tolerated for HrpX-dependent expression (Tsuge et al., 2005), as it was previously demonstrated by cDNA-AFLP and RT-PCR for the *X. euvesicatoria* genes XCV3407 and XCV3765 (Noël et al., 2001; Koebnik et al., 2006). Yet we prefer to use a conservative prediction approach because relaxing the stringency would increase the number of false positives.

**Table 6:** **Presence of consensus PIP-regulated promoter motifs in the ***Xanthomonas*** strains sequenced in this study**.

**Locus**	**Xe 85-10**		**Xe 66b**	**Xe 83M**	**LMG 667**	**LMG 905**	**LMG 909**	**LMG 933**	**LMG 918**	**Xp 91-118**	**Xp 4P1S2**	**R07**
HrpB1	XCV0427	+	+	+	+	+	+	+	+	+	+	+
HrcU	XCV0426	+	+	+	+	+	+	+	+	+	+(ag)	+
HrcQ	XCV0423	+	+	+	+	+	+	+	+	+	+	+
HrcD	XCV0419	+	+	+	+	+	+	+	+	+	+	+
XopA	XCV0440	+	+	+	+	+	+	+	+	+	+	+
HpaH	XCV0441	+	+	+	+	+	+	+	+	+	+	+
XopB	XCV0581	+	+	+	+	+	+	+	+(IS)	no gene	no gene	no gene
XopC1	XCV2435	+(IS)	+(IS)	+(IS)	+(IS)	+(IS)	+(IS)	+(IS)	no gene	no gene	no gene	no gene
XopE1	XCV0294	+	+	+	+	+	+	+	+	+	+	+
XopE2	XCV2280	+	+	+	+	+	+	+	+	no gene	no gene	+
XopJ1	XCV2156	+	+	+	+	+	+	+	+	no gene	no gene	no gene
XopJ2		no gene	no gene	no gene	no gene	no gene	no gene	no gene	no gene	no gene	+	no gene
XopJ4		no gene	no gene	no gene	no gene	no gene	no gene	no gene	no gene	+	+	no gene
XopK	XCV3215	+	+	+	+	+	+	+	+	+	+	+
XopR	XCV0285	+	+	+	+	+	+	+	+	+	+	+
XopAA	XCV3785	+	+	+	+	+	+	+	+(Ψ)	no gene	no gene	no gene
XopAF2		no gene	+	no gene	no gene	no gene	no gene	no gene	+	no gene	no gene	no gene
XopAJ	XCV4428	+	+	+	+	+	+	+	+(IS)	no gene	no gene	+
Endopoly-galacturonase	XCV0722	+	+	+	+	+	+	+	+	+	+	+
Endopoly-galacturonase	XCV2571	+	+	+	+	+	+	+	+	no gene	+	no gene
LipA	XCV0536	+	+	+	+	+	+	+	+	+	+	+
PSP	XCV4424	+	+	+	+	+	+	+	+	+	+	+
PSP	XCV2568	+	+	+	+	+	+	+	+	no gene	+	no gene
YapH	XCV2103	+	+	+	+	+	+	+	+	+	+	+
TrpE	XCV0505	+	+	+	+	+	+	+	+	+	+	+
CHP	XCV2729	+	+	+	+	+	+	+	+	+	+	+
no ORF	α-XCV1852	+	+	+	+	+	+	+	+	no gene	no gene	+
no ORF	α-HP	no gene	no gene	no gene	no gene	no gene	no gene	no gene	no gene	+	+	no gene

HP, Hypothetical protein; CHP, Conserved hypothetical protein; PSP, Putative secreted protein; Ag, assembly gap; Ψ, pseudogene; IS, gene disrupted by an insertion sequence; no gene, ortholog of the gene in strain 85-10 was not detected by BLAST in this strain.

ORFs for two T3 effectors were found downstream of a PIP box-regulated promoter, but are probably no longer under the control of HrpX due to the presence of IS elements. For example, in all the analyzed *X. euvesicatoria* genomes, the *xopC1* ORF starts 1423 bp downstream of the predicted promoter, which suggests that the gene is not HrpX-dependently transcribed in any of the strains due to the polar effects of the IS element. In contrast, an IS element inserted directly behind the—10 promoter motif of *xopB* in LMG 918 likely disrupting gene transcription and/or regulation. These events enlarge the number of T3 effector genes that are in a process of functional erosion, in addition to the cases where IS elements insert into the coding sequence (e.g., *xopJ3, xopO*, and *xopAJ* in strain LMG 918). More experimental work is required to fully elucidate the *hrpX* regulon in the different strains, which might reveal that not only gene repertoires but also gene expression patterns contribute to host and tissue specificity of plant-pathogenic bacteria, such as *Xanthomonas*.

TAL effectors are among the best studied T3 effectors in *Xanthomonas* (Boch and Bonas, 2010). Upon import into the plant cell nucleus, they bind to the DNA in a sequence specific manner and induce transcription of eukaryotic genes in a way that TAL effectors can be considered as trans-kingdom remote controls for gene expression. Their modular structure, however, makes it nearly impossible to assemble TAL genes from short next generation sequencing reads. TBLASTN reads revealed the presence of TAL genes in the LMG strains 667, 909, and 918. Only two TAL effectors have been described and functionally characterized for *X. euvesicatoria*, AvrBs3, and AvrBs4. They are both encoded on plasmids and are extremely similar to each other. Yet, a small indel in the 3′ region of the *avrBs4* gene allows to distinguish them. Based on this polymorphism we predict that LMG 667 and LMG 909 contain an ortholog of *avrBs3* and LMG 918 contains an ortholog of *avrBs4*. We verified a functional copy of *avrBs4* in LMG 918 as it caused HR on tomato and a functional copy of *avrBs3* for LMG 667 and 909 which caused HR on pepper (ECW-30R). This conclusion is further supported by the observation that the upstream and downstream regions are syntenic between strains LMG 667 and 909 and the Macedonian strain 83M, which contains a functional *avrBs3* gene that triggers HR on ECW-30R (*Bs3*) pepper plants but not on ECW plants. Similarly, the upstream region of LMG 918 is syntenic to the corresponding region in the Bulgarian strain 66b, which triggers an HR on the tomato cultivar Moneymaker (*Bs4*) but not in a Moneymaker line with the *bs4* crossed in, and thus contains a functional *avrBs4* gene. BLASTN analyses of the flanking regions of the TAL gene-containing contigs suggests that all *X. euvesicatoria* TAL effectors are encoded on plasmids, including the orthologs that we describe here for the LMG strains.

## Conclusion

This study expands the publicly available genome sequences of *X. euvesicatoria* to include one from each continent where bacterial spot of tomato and pepper exists and from strains isolated in the 1950s and 1970s. Analysis of all the available sequences supports the conclusion that *X. euvesicatoria* and *X. perforans* are one bacterial species. Furthermore, a plethora of bioinformatic data as well as our own analyses supports the designation of all members of Rademaker group 9.2 as *X. euvesicatoria*. We offer direct evidence that *X. euvesicatoria, X. perforans, X. axonopodis* pv. allii, *X. alfalfa* subsp. *citrumelonis*, and *X. dieffenbachiae* belong to the same species, *X. euvesicatoria*. This species should also include the recently described pathogen of rose, herein designated *X. euvesicatoria* pv. rosa. Bioinformatic analysis of whole genomes alone for bacterial phylogeny should be relied upon instead of host range and T3 effectors.

Pathogenicity tests, race analysis, and bioinformatics analysis of T3 effectors are fundamental for the study of host—microbe interaction, but of little relevance to bacterial speciation. Relying on these tests or analyses for phylogeny of bacterial plant pathogens can confuse the concept of bacterial speciation which is now being built on whole genome sequencing. In this study, we inventoried the full repertoire of T3 effectors in sequenced strains of *X. euvesicatoria*. We describe relatively ancient stepwise erosion and acquisition of some T3 effectors. We identified orthologs of *avrBs3* and *avrBs4* highlighting a restriction to host expansion by this pathogen lineage.

## Author contributions

JB and RK conceived the study, analyzed the data, and wrote the manuscript. TV and PL participated in sequencing, assembly and annotation of the LMG strains. JJ, ST, and GV participated in sequencing and annotation of the rose pathogen strain. GM conducted the pathogenicity and biochemical tests. All authors have read and approved the final manuscript.

## Funding

Work in RK's laboratory was supported by the French Agence National de la Recherche (grants ANR-2010-BLAN-1723 and ANR-2010-GENM-013). This work was partially funded by the United States Department of Agriculture, National Institute of Food and Agriculture (USDA-NIFA; grant 2011-670137-30166), and Food Research Institute at the University of Wisconsin—Madison to JB; funds supporting research efforts of GV, JJ, GM, and ST include Specialty Crop Program award #018015 from the Florida Department of Agriculture & Consumer Services, and Specialty Crop Research Initiative award #2015-51181-24312 from the USDA-NIFA. TV thanks the European Union Erasmus+ Program and Campus France for support.

### Conflict of interest statement

The authors declare that the research was conducted in the absence of any commercial or financial relationships that could be construed as a potential conflict of interest.
